# Posttranscriptional Gene Regulation of T Follicular Helper Cells by RNA-Binding Proteins and microRNAs

**DOI:** 10.3389/fimmu.2018.01794

**Published:** 2018-07-31

**Authors:** Dirk Baumjohann, Vigo Heissmeyer

**Affiliations:** ^1^Institute for Immunology, Biomedical Center, Ludwig-Maximilians-Universität München, Planegg-Martinsried, Germany; ^2^Research Unit Molecular Immune Regulation, Helmholtz Zentrum München, Munich, Germany

**Keywords:** T follicular helper, T follicular regulatory, Roquin, regnase-1, microRNAs, miR-17–92, miR-155, miR-146a

## Abstract

T follicular helper (Tfh) cells are critically involved in the establishment of potent antibody responses against infectious pathogens, such as viruses and bacteria, but their dysregulation may also result in aberrant antibody responses that frequently coincide with autoimmune diseases or allergies. The fate and identity of Tfh cells is tightly controlled by gene regulation on the transcriptional and posttranscriptional level. Here, we provide deeper insights into the posttranscriptional mechanisms that regulate Tfh cell differentiation, function, and plasticity through the actions of RNA-binding proteins (RBPs) and small endogenously expressed regulatory RNAs called microRNAs (miRNAs). The Roquin family of RBPs has been shown to dampen spontaneous activation and differentiation of naïve CD4^+^ T cells into Tfh cells, since CD4^+^ T cells with Roquin mutations accumulate as Tfh cells and provide inappropriate B cell help in the production of autoantibodies. Moreover, Regnase-1, an endoribonuclease that regulates a set of targets, which strongly overlaps with that of Roquin, is crucial for the prevention of autoantibody production. Interestingly, both Roquin and Regnase-1 proteins are cleaved and inactivated after TCR stimulation by the paracaspase MALT1. miRNAs are expressed in naïve CD4^+^ T cells and help preventing spontaneous differentiation into effector cells. While most miRNAs are downregulated upon T cell activation, several miRNAs have been shown to regulate the fate of these cells by either promoting (e.g., miR-17–92 and miR-155) or inhibiting (e.g., miR-146a) Tfh cell differentiation. Together, these different aspects highlight a complex and dynamic regulatory network of posttranscriptional gene regulation in Tfh cells that may also be active in other T helper cell populations, including Th1, Th2, Th17, and Treg.

## Introduction

T helper cells are important constituents of the adaptive immune system. They are critically involved in the elimination of various pathogens, including viruses, bacteria, and fungi. Due to their capabilities of forming immunological memory and providing help to B cells, vaccines aim at inducing strong T helper cell responses in concert with cytotoxic T cells and antibody-producing B cells. However, dysregulated T helper cell responses are also associated with several diseases. In allergies, the immune system reacts to normally innocuous compounds of the environment. In autoimmune diseases such as type I diabetes, multiple sclerosis, and rheumatoid arthritis, T helper cells are coordinating critical processes that contribute to tissue inflammation and destruction. In cancer, dysregulated T helper cells might on the one hand be impaired in their proper functioning, thus limiting the body’s immune response against neoplastic cells and tissues. On the other hand, hyper-responsiveness or malignant transformation of T helper cells can drive chronic inflammation or induce neoplasia, respectively.

T helper cells comprise many different subsets that are each tailored to the functional response that these cells elicit against various different pathogens. The major T helper cell subsets include Th1, Th2, Th17, T follicular helper (Tfh), and regulatory T (Treg) cells, which can be differentiated by their characteristic expression of signature transcription factors, chemokine receptors, and cytokines (1). While initially it was believed that Th2 cells provide help to B cells, it is now accepted that Tfh cells are the major subset of T helper cells that is specialized in providing help to B cells for the establishment and maintenance of germinal centers (GCs) and for the production of high-affinity antibodies (2–4). In line with this, Tfh cells express the chemokine receptor CXCR5, which facilitates the migration of activated CD4^+^ T cells to the T-B zone border and further into the B cell follicle. This aspect also reflects the step-wise differentiation process of Tfh cells, which are initially primed by dendritic cells, followed by sequential interactions with activated B cells and GC B cells (2–4). Tfh cells produce various cytokines, including IL-21 and IL-4, and they express several costimulatory molecules, including ICOS, CD40L, and PD-1, which allow for reciprocal interactions with B cells. Tfh cells are further characterized by the expression of the transcription factors Ascl2 and Bcl6, and the adaptor molecule SAP. Besides Tfh cells, T follicular regulatory (Tfr) cells have been identified as a hybrid cell population of Tfh and Treg cells that prevent excessive humoral immune responses (5). They express the signature transcription factors of Tfh and Treg cells, Bcl6 and Foxp3, respectively, and share additional characteristics of both T helper cell subsets.

The differentiation of naïve CD4^+^ T cells into effector and memory cells is tightly regulated on the molecular level (6–8). Several signature or “master” transcription factors have been identified that are specific for the respective T helper cell subset, e.g., T-bet for Th1, Gata-3 for Th2, RORγt for Th17, Bcl6 for Tfh, and Foxp3 for Treg. Often, these transcription factors also inhibit each other’s function, thus contributing to cell fate decisions of the differentiating cells. Upstream of these “lineage”-defining transcription factors, combinations of Jak (Janus kinase) and signal transducer and activator of transcription (STAT) molecules that transduce signaling events from cytokine receptors have also been associated with the different T helper cell populations (9). Given the variety of T helper cell qualities, gene expression needs to be thoroughly regulated in activated CD4^+^ T cells to ensure proper differentiation into the different T helper cell subsets (10). Beside the direct transcriptional regulation through STATs and other transcription factors, transcribed mRNAs are furthermore highly regulated on the posttranscriptional level. Several mechanisms contribute to this regulation, including RNA-binding proteins (RBPs) and microRNAs (miRNAs), which can act cooperatively on different as well as on similar molecular pathways. In this review, we discuss the role of different RBPs and miRNAs in shaping Tfh cell identity and function.

## Posttranscriptional Gene Regulation by RBPs in T Cells

RNA-binding proteins are *trans*-acting factors that interact with specific *cis*-elements in RNAs by recognizing linear sequence motifs or dynamically forming secondary structures. The binding to *cis*-elements in the 5′ UTR typically controls translation initiation, while binding to sites in the 3′ UTRs of transcripts typically regulates mRNA decay or translation efficiency (11).

The Roquin family of RBPs includes the paralogs Roquin-1 and Roquin-2. These proteins are encoded by the *Rc3h1* and *Rc3h2* genes and serve redundant functions in T cells (12–14). The Regnase family comprises the paralogs Regnase-1, Regnase-2, Regnase-3, and Regnase-4 also known as Mcpip1, 2, 3, and 4, which are encoded by the *Zc3h12a, Zc3h12b, Zc3h12c*, and *Zc3h12d* genes (15). The redundancy of Regnase proteins has not been addressed experimentally; however, Regnase-1 and Regnase-4 proteins appear to be the T cell-expressed paralogs (15). Regnase-1 and Roquin proteins predominantly bind to 3′ UTRs of mRNAs (16, 17) and play important roles in the regulation of T cell fate decisions (14, 18–22). Roquin proteins recognize stem-loop structures of the tri- or hexa-loop containing CDE or ADE consensus motifs, respectively (17, 23–30). These interactions allow the recruitment of mRNA degrading enzymes (24, 31, 32) and induce decay of target mRNAs. Regnase-1 also appears to repress targets through similar stem-loop structures (16, 21, 33, 34) that are present in an overlapping set of target mRNAs with pro-inflammatory functions (16, 20). However, the endonuclease Regnase-1 may rather cleave target mRNAs itself or, dependent on the 3′ UTR, induce translational inhibition (16, 21, 33–35). Among the well-established targets of Roquin and Regnase proteins are *ICOS, Ox40, Il6, cRel, Irf4, Nfkbiz*, and *Nfkbid* (14, 16–24, 28, 33, 34). Interestingly, the mRNAs encoding for Roquin and Regnase proteins themselves contain *cis*-elements that enable the system to fine-tune expression levels through negative autoregulation (18, 20, 24, 27, 33). However, it is currently under debate whether these factors cooperate in posttranscriptional gene regulation or work independently in a spatially and temporally compartmentalized fashion (16, 20, 36, 37).

**Figure 1:**
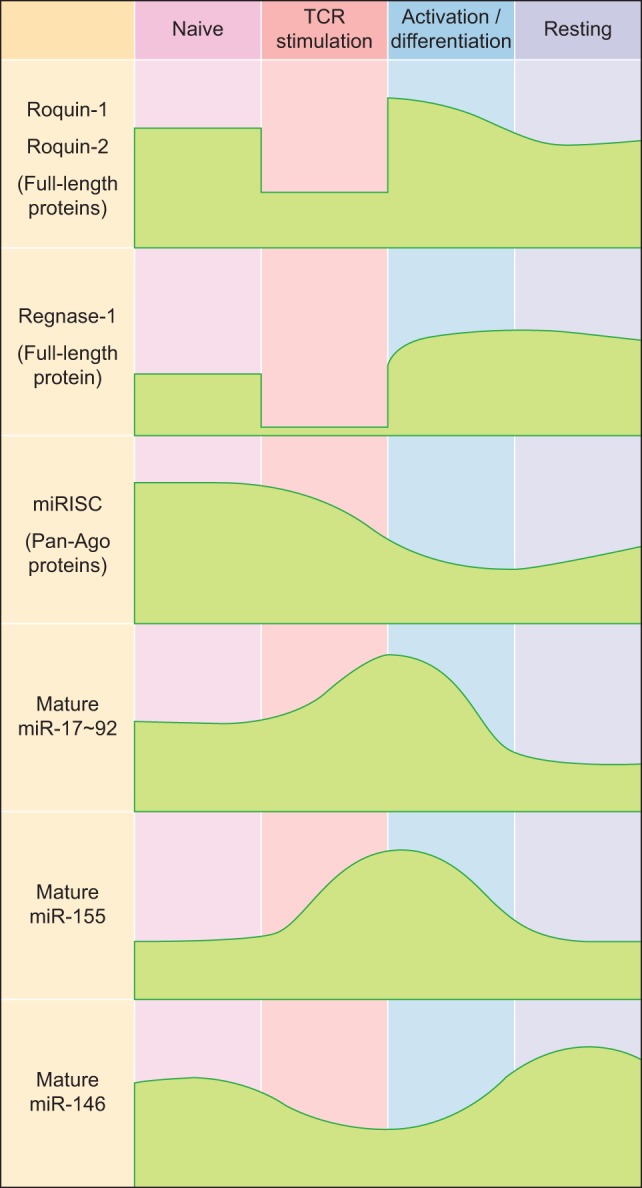
Expression kinetics of major RNA-binding proteins and microRNAs (miRNAs) during T helper cell differentiation. Abbreviations: miRISC, miRNA-induced silencing complex; Ago, Argonaute.

While Roquin-1 and the less abundant Roquin-2 proteins show rather constitutive expression in T cells (14) and are only moderately upregulated in response to TCR-dependent T cell stimulation (38), the most prominent member of the Regnase family of proteins in T cells, Regnase-1, is weakly expressed in naive T cells, but becomes induced during TCR-dependent activation of T cells (39) (Figure 1). However, during TCR signaling itself, Roquin-1 and Roquin-2 as well as Regnase-1 proteins are cleaved and functionally inactivated by the MALT1 paracaspase (20, 21, 40) (Figure 1).

## RNA-Binding Protein-Mediated Regulation of Tfh and Tfr Cells

**Figure 2:**
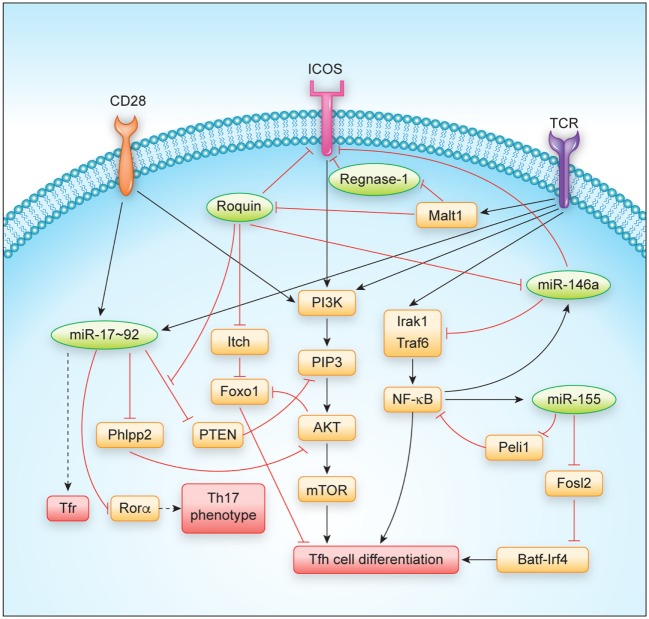
Regulation of T follicular helper cell differentiation and function by major RNA-binding proteins and microRNAs. Abbreviations: TCR, T cell receptor.

The Roquin and Regnase-1 RBPs have been shown to be involved in the regulation of the GC reaction and prevention of autoimmunity, since mutation or loss-of-function of the encoding genes lead to spontaneous activation of T cells and the development of antinuclear antibodies in mice (14, 18, 20–22). Their role in Tfh and Tfr cells will be described in more detail here (Figure 2).

## Roquin

The gene encoding for Roquin-1 was identified in the lab of Christopher Goodnow by screening of mice for ethyl nitroso urea-induced mutations that caused the formation of antinuclear autoantibodies (22). Homozygous mutation exchanging one single amino acid of M199R in Roquin-1, as determined in the so-called *sanroque* mouse strain, was found to cause a dramatic activation of CD8^+^ and CD4^+^ T cells and led to the accumulation of Tfh cells. Spleens of these mice contained large numbers of GCs and the induced GC B cells produced high-affinity antibodies against a large variety of self-antigens (22, 41). Surprisingly, the knockout of the Roquin-1-encoding gene *Rc3h1* showed postnatal lethality and mild immune dysregulation but did not recapitulate the flagrant autoimmune phenotype of *sanroque* mice (42). Nevertheless, combined deletion of Roquin-1 and Roquin-2 encoding genes in T cells resulted in the spontaneous activation of CD4^+^ and CD8^+^ T cells and the accumulation of Tfh cells and GC B cells. These findings demonstrated redundant functions of both proteins in T cells and suggested a compensatory function of the much lower expressed Roquin-2 protein in the absence of Roquin-1, but not when Roquin-1^san^ protein is expressed (14). In mice lacking Roquin-1 and Roquin-2-encoding alleles in T cells, the splenic architecture was greatly disturbed and, as a probable consequence, less self-reactive antibodies were observed in the sera (14, 20).

The molecular mechanisms underlying spontaneous T cell activation and Tfh cell differentiation are likely to involve several Roquin-regulated targets that synergize in this differentiation program. Initially, the dysregulation of ICOS, the first and best-studied Roquin target (22, 28, 31, 38, 43, 44), was proposed to explain the observed autoimmune phenotype (45). However, *sanroque* mice that were additionally deficient in *Icos* were later shown to maintain many phenotypes including Tfh cell accumulation (46). Instead, accumulation of Tfh cells in *sanroque* mice was a consequence of the excessive production of IFN-γ that occurs in these mice, as was demonstrated in combination of *sanroque* and IFN-γ receptor (*Ifngr*) knockout genotypes (46). At this point, it is not clear how IFN-γ becomes induced in *sanroque* mice, since the *Ifng* mRNA is rather strongly regulated by AU-rich elements (AREs), which are recognized by ARE-binding proteins like TTP, AUF, or HUR proteins, and genetic deletion of these AREs has been demonstrated to also cause a lupus-like phenotype in mice (47, 48). As compared to *sanroque* mice, CD4^+^ T cells lacking Roquin proteins also did not show a similarly strong Th1 bias, but rather differentiated into Th17 cells *in vitro*, a phenotype that developed in addition to the shared spontaneous differentiation into Tfh cells (20). This differential bias may relate to a partial or complete derepression of the different Roquin-regulated targets including *ICOS, Irf4, cRel, Nfkbiz*, and *Nfkbid* that have been shown to affect Tfh as well as Th17 differentiation (49–58). One key signaling cascade influenced by Roquin has been identified in the PI3K-Akt-mTOR and Foxo1 pathway in which Roquin regulates the expression of *ICOS, Pten*, and *Itch* mRNAs (19, 31, 44) (Figure 2). The *ICOS* and *Itch* mRNAs are bound and negatively regulated, leading to increased ICOS and Itch levels in the absence of Roquin (19, 28, 31, 38). Increased ICOS expression and signaling stimulates PIP3 formation that activates the kinase Akt, which phosphorylates and thereby inactivates Foxo1, a transcription factor that strongly inhibits Tfh differentiation (57). In contrast, elevation of Itch, a Foxo1-specific E3 ubiquitin ligase, decreases cellular levels of Foxo1 (57, 59). Different from the other Roquin targets, Pten levels decreased in CD4^+^ T cells and Treg cells upon induced ablation of Roquin-encoding alleles (19). Interestingly, the Roquin-bound sequences in the 3′ UTR of *Pten* showed conservation of those nucleotides that were involved in forming a stem-loop (19), but at the same time overlapped with a miR-17 binding site, which was previously shown to effectively regulate Pten levels in T cells (60). Biochemical evidence showed that Ago2 more efficiently associated with *Pten* mRNA in the absence of Roquin (19), suggesting a structure switch mode within this *cis*-element and a competitive interaction and regulation of *Pten* mRNA by Roquin and miR-17–92 containing RNA-induced silencing complex (miRISC) transacting factors. The regulation of PI3K-Akt-mTOR and Foxo1 signaling through Roquin-mediated regulation of ICOS, Itch, and Pten targets not only contributes to the observed skewing of T cell differentiation into Tfh and Th17 and against induced Treg (iTreg) cell differentiation, but also correlates with a conversion of thymus-derived Treg cells into Tfr cells *in vivo* (19). Roquin-deficient Treg cells lost CD25 expression, upregulated a Tfh but downregulated their Treg gene signature and retained their ability to control antigen-dependent GC B cell responses and affinity maturation of antibodies. In contrast, Roquin-deficient Treg cells were less able to prevent spontaneous activation of CD4^+^ and CD8^+^ T cells and to protect from T cell transfer-induced colitis (19).

## Regnase-1

Regnase-1 was initially described as an LPS-induced gene and the knockout of Regnase-1 caused a severe auto-inflammatory phenotype in mice. The molecular basis for this phenotype was proposed to involve Regnase-1-dependent regulation of IL-6 and IL-12p40 regulation in myeloid cells (34). However, more recently, it was shown that the combined knockout of Regnase-1 with IL-6 or IL-12-encoding alleles did not fully rescue central phenotypes. Instead, conditional T cell-specific deletion of Regnase-1 phenocopied most of the phenotypes of the global Regnase-1 knockout (21). The consequences of Regnase-1 deficiency for Tfh differentiation have not been experimentally addressed so far, but the following observations could argue for a control of Tfh differentiation by Regnase-1: First, upon genetic inactivation of Regnase-1 globally or specifically in T cells, mice develop autoantibodies and show elevated plasma cell levels as well as an accumulation of all immunoglobulin isotypes in their sera (21, 34). Second, at least for the regulation of a CDE-containing element in the 3′ UTR of the *Tnf* mRNA, Regnase-1 has been demonstrated to functionally cooperate with Roquin in target regulation, and this mode of direct or indirect interaction may apply to several other shared target mRNAs that have an effect on Tfh cell differentiation (20, 37). Moreover, the systemic IFN-γ production that was found to drive Tfh cell differentiation in *sanroque* mice (46) was similarly observed upon deletion of Regnase-1-encoding alleles in T cells (18, 21). Finally, among the targets that have been reported to be regulated by Regnase-1 are several gene products that are known to promote Tfh differentiation, including ICOS, Ox40, and IL-6 (2, 61). Future experiments should demonstrate how Regnase-1 or other Regnase paralogs affect Tfh cell differentiation in a T cell-intrinsic or extrinsic manner.

## Posttranscriptional Gene Regulation by miRNAs in T Cells

MicroRNAs are small endogenously expressed RNAs that regulate gene expression. Each miRNA can have several hundred target genes and a given mRNA might in turn be regulated by many different miRNAs simultaneously. These features of miRNAs result in redundancy that is believed to buffer gene expression and to confer biological robustness (62–64). In line with this, miRNAs and the miRNA-induced silencing complex (miRISC) are relatively highly expressed in naïve CD4^+^ T cells, thereby contributing to the prevention of spontaneous differentiation into effector cells (65, 66) (Figure 1). In activated T cells, most miRNAs are downregulated, while only a few so-called driver-miRNAs are differentially upregulated to act in concert with transcription factors for proper T helper cell differentiation and function (67–71) (Figure 1). Initial experiments that utilized genetically engineered mice in which T cells lacked mature miRNAs due to ablation of miRNA-processing proteins such as Dicer established functional roles for miRNAs in the generation of Th1, Th2, Th17, and Treg cells (72, 73). While all these T helper cell populations could still be generated to various degrees from miRNA-deficient naïve CD4^+^ precursor cells, global miRNA expression in CD4^+^ T cells was absolutely required for the differentiation of naïve CD4^+^ T cells into mature Tfh cells *in vivo* (74). To date, various T cell-expressed miRNAs and miRNA clusters have been shown to play critical roles in the differentiation and function of Tfh cells (69) and in the establishment and maintenance of GCs (75), and these findings indicate that Tfh cells may be particularly sensitive to the regulation by miRNAs.

## miRNA-Mediated Regulation of Tfh and Tfr Cells

Among the individual miRNAs that have been studied in the context of Tfh cells, the function of the miR-17–92 cluster and the miR-155/miR-146a axis have been investigated in most detail and will be described here (Figure 2).

## The miR-17–92 Cluster

The miR-17–92 cluster consists of six individual miRNAs that can be grouped into four distinct miRNA families according to their seed sequences (76). Even though miR-17–92 is transcribed as a common transcript and highly induced in activated CD4^+^ T cells (65, 77–80) (Figure 1), the individual miRNA cluster members are differentially processed thereafter (81). miR-17–92 is not only critically involved in the regulation of Tfh cells (as discussed in more detail below), but it is also important for the differentiation and function of other T helper cell subsets [reviewed in Ref. (82)], including Th1 (80, 83), Treg (77, 83), Th2 (84), and Th17 cells (79, 85). Interestingly, miR-17–92 shares several features between Th2 cells and type 2 innate lymphoid cells (86), indicating that many of the miR-17–92 cluster’s functions may be conserved between the individual T helper cell subsets and their respective ILC counterparts. First evidence for a role of miR-17–92 in Tfh cells (Figure 1) came from an early study in which Bcl6 overexpression resulted in reduced miR-17–92 expression, with miR-17–92 itself repressing CXCR5 (87). However, more recent studies clarified the Tfh-promoting function of miR-17–92 (74, 78, 80). Deletion of the miR-17–92 cluster in T cells resulted in reduced Tfh cell differentiation, whereas transgenic overexpression of the cluster resulted in higher frequencies and numbers of Tfh cells. On the mechanistic level, miR-17–92 was found to target *Pten* and *Phlpp2*, a phosphatase in the ICOS signaling pathway (74, 78). Besides these Tfh-promoting effects, miR-17–92 also prevented the expression of genes that are normally not associated with Tfh cells during LCMV infection, but instead are usually associated with Th17 cells, including *Ccr6, Rora, Il22, Il1r1*, and *Il1r2* (74). Importantly, it was further shown that each miRNA of the miR-17–92 cluster directly targeted the *Rora* 3′ UTR and that this axis contributed to repressing the Th17-associated gene expression program in wild-type Tfh cells (74). Since most of these experiments were performed with mice deficient in or overexpressing the entire miR-17–92 cluster in T cells *in vivo*, not much is known about the contribution of the individual miRNAs of this cluster to Tfh cell differentiation and function. This would be important though, because individual miRNAs of this cluster can have cooperative but also opposing effects on T cells, which is further amplified by the complexity of the downstream target gene networks. The continuing lack of reliable protocols for the *in vitro* differentiation of murine Tfh cells (2) currently impairs the ability to perform *in vitro* experiments that interrogate individual miRNA functions specifically in mouse Tfh cells. Nevertheless, recent technological advances such as CRISPR/Cas9 or mouse lines that lack individual cluster members (88) might substitute for this current limitation. Using human Tfh cell *in vitro* cultures, a recent report found that miR-92a targets *KLF2* and *PTEN*, thereby promoting Tfh cell differentiation (89). Similar to Tfh cells, Tfr cells are also responsive to the dose of miR-17–92 regulation (74). As a first example of how RBPs can intersect with miRNA-dependent gene regulation of Tfh differentiation, it was recently shown that Roquin (see above) interferes with miR-17–92 binding to an overlapping *cis*-element in the *Pten* 3′ UTR, which leads to inhibition of the PI3K–Akt–mTOR signaling pathway, thereby inhibiting the conversion of Treg to Tfr cells (19).

## The miR-155/miR-146a Axis

Similar to miR-17–92, miR-155 is induced and highly expressed in activated T helper cells (65, 90–93) (Figure 1). miR-155 has been shown to be important for proper differentiation of Th1 and Th17 cells and for EAE pathogenesis (94–96), as well as for Treg differentiation and function (97, 98). In contrast to miR-155, miR-146a is highly expressed in naïve CD4^+^ T cells and initially downregulated in activated T cells (65, 90) (Figure 1). miR-146a is subsequently upregulated in differentiating Tfh cells (55), reaching the highest expression levels among hematopoietic cells in mature Tfh and GC B cells (99). In comparison to the total numbers of Tfh cells elicited during an immune response, the kinetics of increased miR-146a expression slightly lagged behind (55). These data indicate that miR-146a acts as a negative regulator of Tfh cells that prevents excessive Tfh cell numbers and thereby limits GC responses (69). In T cells, an epistatic relationship between miR-146a and miR-155 has been described in which miR-155 promotes and miR-146 inhibits IFNγ responses (100). Further studies have also established a role for miR-146a in T cell activation (101, 102) as well as in differentiation and function of different T helper cell subsets, including Th1, Th17, and Treg cells (103–106). miR-146a-deficient mice develop a chronic inflammatory phenotype with progressive myeloproliferation and eventually myeloid and lymphoid malignancies (107). In these mice, Tfh cells accumulate due to the Tfh cell-promoting function of miR-155 (see below), thus further highlighting the reciprocal regulation of Tfh cell differentiation by these two miRNAs (108). Mechanistically, miR-146a was found to repress several Tfh-associated genes, including ICOS, which was highly upregulated in miR-146a-deficient CD4^+^ T cells (55). miR-146a itself is also regulated by Roquin (43). In a different study, it was shown that miR-155 promoted Tfh cell differentiation by repressing the expression of *Peli1*, a ubiquitin ligase that promotes the degradation of the NF-κB family transcription factor c-Rel, which itself controls cellular proliferation and CD40L expression (109). Another study found that miR-155 expression in hematopoietic cells was required for the differentiation of Tfh and GC B cells following murine gammaherpesvirus infection (110). Together, these data indicate a tightly controlled reciprocal function of miR-155 and miR-146a in the regulation of Tfh cell differentiation and function.

## Conclusion

T follicular helper cells require continuous stimulation (111, 112) and the differentiation of these cells is more dependent on costimulatory signals than other T helper cell subsets (113). This might be the reason for why they are so responsive to the regulation by RBPs and miRNAs. Both classes of trans-acting factors cooperate to shape the expression levels not only of costimulatory molecules but also of intracellular transducers of signals in the PI3K-Akt-mTOR and Foxo1 pathway (Figure 2). PI3K activity is strongly stimulated by ICOS co-stimulation as well as TCR signaling and was shown to drive the Tfh differentiation program (114). Besides the well-established miRNA regulators of Tfh cells, the miR-17–92 cluster and the miR-155/miR-146a axis, other miRNAs may also play important roles in Tfh cell differentiation and T helper cell plasticity. For example, miR-10a is highly expressed in Treg cells (99, 115, 116). TGF-beta induced miR-10a represses the Tfh-associated transcriptional repressor *Bcl6*, thereby preventing the conversion of Treg cells into Tfh cells (116). In human Tfh cells, miR-31 was recently shown to be downregulated by BCL6, thereby resulting in the upregulation of the miR-31 target mRNAs encoding the Tfh-associated molecules CD40L, SAP, and BTLA (117). The finding that Tfh cell differentiation is completely blocked in miRNA-deficient CD4^+^ T cells (74) further indicates that additional miRNAs must play important roles in Tfh cell biology. Moreover, it remains largely unclear in how far miRNAs and their target gene networks contribute to the function and maintenance of Tfh cells. It is also likely that upon comprehensive identification of RBPs that are bound to mRNAs in T cells and upon individual testing of these factors, additional contributions of RBPs in T cell differentiation will be uncovered. Such future investigations will provide novel insights into how the loss of individual miRNAs or individual RBPs affects T cell differentiation programs. Additional contributions may result from other levels of posttranscriptional regulation such as alternative splicing, alternative polyadenylation, or RNA modifications, which have already been shown to regulate Tfh-relevant genes (118–120), albeit, it has not yet been tested how these processes may impact Tfh differentiation. Another big task will be to understand how simultaneous inputs from different posttranscriptional regulators generate a specific response. Here, it will be critical to comprehensively identify and dissect all *cis*-elements encoded in mRNAs with strong impact on T cell differentiation programs (Figure 2). These analyses should be combined with structural and biochemical information to integrate the emerging evidence of RNA-modifications, to describe dynamics of RNA/protein-complex formation, and to understand how the individual binding sites act redundantly, cooperatively, antagonistically, or synergistically in posttranscriptional gene regulation, thus enabling cell-fate decisions.

## Author Contributions

Both authors contributed to conceptualizing and writing the manuscript.

## Conflict of Interest Statement

The authors declare that the research was conducted in the absence of any commercial or financial relationships that could be construed as a potential conflict of interest.

## References

[1] Sallusto F. Heterogeneity of human CD4(+) T cells against microbes. Annu Rev Immunol (2016) 34:317–34. 10.1146/annurev-immunol-032414-112056 27168241

[2] Crotty S. T follicular helper cell differentiation, function, and roles in disease. Immunity (2014) 41:529–42. 10.1016/j.immuni.2014.10.004 25367570 PMC4223692

[3] Qi H. T follicular helper cells in space-time. Nat Rev Immunol (2016) 16(10):612–25. 10.1038/nri.2016.94 27573485

[4] Vinuesa CG Linterman MA Yu D MacLennan IC. Follicular helper T cells. Annu Rev Immunol (2016) 34:335–68. 10.1146/annurev-immunol-041015-055605 26907215

[5] Sage PT Sharpe AH. T follicular regulatory cells. Immunol Rev (2016) 271:246–59. 10.1111/imr.12411 27088919

[6] DuPage M Bluestone JA. Harnessing the plasticity of CD4(+) T cells to treat immune-mediated disease. Nat Rev Immunol (2016) 16:149–63. 10.1038/nri.2015.18 26875830

[7] O’Shea JJ Paul WE. Mechanisms underlying lineage commitment and plasticity of helper CD4+ T cells. Science (2010) 327:1098–102. 10.1126/science.1178334 20185720 PMC2997673

[8] Zhu J Yamane H Paul WE. Differentiation of effector CD4 T cell populations (*). Annu Rev Immunol (2010) 28:445–89. 10.1146/annurev-immunol-030409-101212 20192806 PMC3502616

[9] O’Shea JJ Schwartz DM Villarino AV Gadina M McInnes IB Laurence A. The JAK-STAT pathway: impact on human disease and therapeutic intervention. Annu Rev Med (2015) 66:311–28. 10.1146/annurev-med-051113-024537 25587654 PMC5634336

[10] Fang D Zhu J. Dynamic balance between master transcription factors determines the fates and functions of CD4 T cell and innate lymphoid cell subsets. J Exp Med (2017) 214:1861–76. 10.1084/jem.20170494 28630089 PMC5502437

[11] Rissland OS. The organization and regulation of mRNA-protein complexes. Wiley Interdiscip Rev RNA (2017) 8(1):e1369. 10.1002/wrna.1369 27324829 PMC5213448

[12] Heissmeyer V Vogel KU. Molecular control of Tfh-cell differentiation by Roquin family proteins. Immunol Rev (2013) 253:273–89. 10.1111/imr.12056 23550652

[13] Pratama A Ramiscal RR Silva DG Das SK Athanasopoulos V Fitch J Roquin-2 shares functions with its paralog Roquin-1 in the repression of mRNAs controlling T follicular helper cells and systemic inflammation. Immunity (2013) 38:669–80. 10.1016/j.immuni.2013.01.011 23583642

[14] Vogel KU Edelmann SL Jeltsch KM Bertossi A Heger K Heinz GA Roquin paralogs 1 and 2 redundantly repress the Icos and Ox40 costimulator mRNAs and control follicular helper T cell differentiation. Immunity (2013) 38:655–68. 10.1016/j.immuni.2012.12.004 23583643

[15] Akira S. Regnase-1, a ribonuclease involved in the regulation of immune responses. Cold Spring Harb Symp Quant Biol (2013) 78:51–60. 10.1101/sqb.2013.78.019877 24163394

[16] Mino T Murakawa Y Fukao A Vandenbon A Wessels HH Ori D Regnase-1 and Roquin regulate a common element in inflammatory mRNAs by spatiotemporally distinct mechanisms. Cell (2015) 161:1058–73. 10.1016/j.cell.2015.04.029 26000482

[17] Murakawa Y Hinz M Mothes J Schuetz A Uhl M Wyler E RC3H1 post-transcriptionally regulates A20 mRNA and modulates the activity of the IKK/NF-kappaB pathway. Nat Commun (2015) 6:7367 10.1038/ncomms8367 26170170 PMC4510711

[18] Cui X Mino T Yoshinaga M Nakatsuka Y Hia F Yamasoba D Regnase-1 and Roquin nonredundantly regulate Th1 differentiation causing cardiac inflammation and fibrosis. J Immunol (2017) 199:4066–77. 10.4049/jimmunol.1701211 29127149

[19] Essig K Hu D Guimaraes JC Alterauge D Edelmann S Raj T Roquin suppresses the PI3K-mTOR signaling pathway to inhibit T helper cell differentiation and conversion of Treg to Tfr cells. Immunity (2017) 47:1067–1082.e1012. 10.1016/j.immuni.2017.11.008 29246441

[20] Jeltsch KM Hu D Brenner S Zoller J Heinz GA Nagel D Cleavage of roquin and regnase-1 by the paracaspase MALT1 releases their cooperatively repressed targets to promote T(H)17 differentiation. Nat Immunol (2014) 15:1079–89. 10.1038/ni.3008 25282160

[21] Uehata T Iwasaki H Vandenbon A Matsushita K Hernandez-Cuellar E Kuniyoshi K Malt1-induced cleavage of regnase-1 in CD4(+) helper T cells regulates immune activation. Cell (2013) 153:1036–49. 10.1016/j.cell.2013.04.034 23706741

[22] Vinuesa CG Cook MC Angelucci C Athanasopoulos V Rui L Hill KM A RING-type ubiquitin ligase family member required to repress follicular helper T cells and autoimmunity. Nature (2005) 435:452–8. 10.1038/nature03555 15917799

[23] Janowski R Heinz GA Schlundt A Wommelsdorf N Brenner S Gruber AR Roquin recognizes a non-canonical hexaloop structure in the 3’-UTR of Ox40. Nat Commun (2016) 7:11032. 10.1038/ncomms11032 27010430 PMC5603727

[24] Leppek K Schott J Reitter S Poetz F Hammond MC Stoecklin G. Roquin promotes constitutive mRNA decay via a conserved class of stem-loop recognition motifs. Cell (2013) 153:869–81. 10.1016/j.cell.2013.04.016 23663784

[25] Oikonomou P Goodarzi H Tavazoie S. Systematic identification of regulatory elements in conserved 3’ UTRs of human transcripts. Cell Rep (2014) 7:281–92. 10.1016/j.celrep.2014.03.001 24656821 PMC4430845

[26] Ramanathan M Majzoub K Rao DS Neela PH Zarnegar BJ Mondal S RNA-protein interaction detection in living cells. Nat Methods (2018) 15:207–12. 10.1038/nmeth.4601 29400715 PMC5886736

[27] Sakurai S Ohto U Shimizu T. Structure of human Roquin-2 and its complex with constitutive-decay element RNA. Acta Crystallogr F Struct Biol Commun (2015) 71:1048–54. 10.1107/S2053230X15011887 26249698 PMC4528940

[28] Schlundt A Heinz GA Janowski R Geerlof A Stehle R Heissmeyer V Structural basis for RNA recognition in roquin-mediated post-transcriptional gene regulation. Nat Struct Mol Biol (2014) 21:671–8. 10.1038/nsmb.2855 25026077

[29] Schuetz A Murakawa Y Rosenbaum E Landthaler M Heinemann U. Roquin binding to target mRNAs involves a winged helix-turn-helix motif. Nat Commun (2014) 5:5701. 10.1038/ncomms6701 25504471

[30] Tan D Zhou M Kiledjian M Tong L. The ROQ domain of Roquin recognizes mRNA constitutive-decay element and double-stranded RNA. Nat Struct Mol Biol (2014) 21:679–85. 10.1038/nsmb.2857 25026078 PMC4125485

[31] Glasmacher E Hoefig KP Vogel KU Rath N Du L Wolf C Roquin binds inducible costimulator mRNA and effectors of mRNA decay to induce microRNA-independent post-transcriptional repression. Nat Immunol (2010) 11:725–33. 10.1038/ni.1902 20639877

[32] Sgromo A Raisch T Bawankar P Bhandari D Chen Y Kuzuoglu-Ozturk D A CAF40-binding motif facilitates recruitment of the CCR4-NOT complex to mRNAs targeted by *Drosophila* Roquin. Nat Commun (2017) 8:14307. 10.1038/ncomms14307 28165457 PMC5303829

[33] Iwasaki H Takeuchi O Teraguchi S Matsushita K Uehata T Kuniyoshi K The IkappaB kinase complex regulates the stability of cytokine-encoding mRNA induced by TLR-IL-1R by controlling degradation of regnase-1. Nat Immunol (2011) 12:1167–75. 10.1038/ni.2137 22037600

[34] Matsushita K Takeuchi O Standley DM Kumagai Y Kawagoe T Miyake T Zc3h12a is an RNase essential for controlling immune responses by regulating mRNA decay. Nature (2009) 458:1185–90. 10.1038/nature07924 19322177

[35] Behrens G Winzen R Rehage N Dorrie A Barsch M Hoffmann A A translational silencing function of MCPIP1/Regnase-1 specified by the target site context. Nucleic Acids Res (2018) 46:4256–70. 10.1093/nar/gky106 29471506 PMC5934641

[36] Fu M Blackshear PJ. RNA-binding proteins in immune regulation: a focus on CCCH zinc finger proteins. Nat Rev Immunol (2017) 17:130–43. 10.1038/nri.2016.129 27990022 PMC5556700

[37] Jeltsch KM Heissmeyer V. Regulation of T cell signaling and autoimmunity by RNA-binding proteins. Curr Opin Immunol (2016) 39:127–35. 10.1016/j.coi.2016.01.011 26871597

[38] Rehage N Davydova E Conrad C Behrens G Maiser A Stehklein JE Binding of NUFIP2 to Roquin promotes recognition and regulation of ICOS mRNA. Nat Commun (2018) 9:299. 10.1038/s41467-017-02582-1 29352114 PMC5775257

[39] Li M Cao W Liu H Zhang W Liu X Cai Z MCPIP1 down-regulates IL-2 expression through an ARE-independent pathway. PLoS One (2012) 7:e49841. 10.1371/journal.pone.0049841 23185455 PMC3504106

[40] Gewies A Gorka O Bergmann H Pechloff K Petermann F Jeltsch KM Uncoupling Malt1 threshold function from paracaspase activity results in destructive autoimmune inflammation. Cell Rep (2014) 9:1292–305. 10.1016/j.celrep.2014.10.044 25456129

[41] Linterman MA Rigby RJ Wong RK Yu D Brink R Cannons JL Follicular helper T cells are required for systemic autoimmunity. J Exp Med (2009b) 206:561–76. 10.1084/jem.20081886 19221396 PMC2699132

[42] Bertossi A Aichinger M Sansonetti P Lech M Neff F Pal M Loss of Roquin induces early death and immune deregulation but not autoimmunity. J Exp Med (2011) 208:1749–56. 10.1084/jem.20110578 21844204 PMC3171092

[43] Srivastava M Duan G Kershaw NJ Athanasopoulos V Yeo JH Ose T Roquin binds microRNA-146a and Argonaute2 to regulate microRNA homeostasis. Nat Commun (2015) 6:6253. 10.1038/ncomms7253 25697406 PMC4346627

[44] Yu D Tan AH Hu X Athanasopoulos V Simpson N Silva DG Roquin represses autoimmunity by limiting inducible T-cell co-stimulator messenger RNA. Nature (2007) 450:299–303. 10.1038/nature06253 18172933

[45] Linterman MA Rigby RJ Wong R Silva D Withers D Anderson G Roquin differentiates the specialized functions of duplicated T cell costimulatory receptor genes CD28 and ICOS. Immunity (2009) 30:228–41. 10.1016/j.immuni.2008.12.015 19217324

[46] Lee SK Silva DG Martin JL Pratama A Hu X Chang PP Interferon-gamma excess leads to pathogenic accumulation of follicular helper T cells and germinal centers. Immunity (2012) 37:880–92. 10.1016/j.immuni.2012.10.010 23159227

[47] Hodge DL Berthet C Coppola V Kastenmuller W Buschman MD Schaughency PM IFN-gamma AU-rich element removal promotes chronic IFN-gamma expression and autoimmunity in mice. J Autoimmun (2014) 53:33–45. 10.1016/j.jaut.2014.02.003 24583068 PMC4148478

[48] Villarino AV Katzman SD Gallo E Miller O Jiang S McManus MT Posttranscriptional silencing of effector cytokine mRNA underlies the anergic phenotype of self-reactive T cells. Immunity (2011) 34:50–60. 10.1016/j.immuni.2010.12.014 21236706 PMC3955755

[49] Annemann M Wang Z Plaza-Sirvent C Glauben R Schuster M Ewald Sander F IkappaBNS regulates murine Th17 differentiation during gut inflammation and infection. J Immunol (2015) 194:2888–98. 10.4049/jimmunol.1401964 25694610

[50] Brustle A Heink S Huber M Rosenplanter C Stadelmann C Yu P The development of inflammatory T(H)-17 cells requires interferon-regulatory factor 4. Nat Immunol (2007) 8:958–66. 10.1038/ni1500 17676043

[51] Chen G Hardy K Pagler E Ma L Lee S Gerondakis S The NF-kappaB transcription factor c-Rel is required for Th17 effector cell development in experimental autoimmune encephalomyelitis. J Immunol (2011) 187:4483–91. 10.4049/jimmunol.1101757 21940679

[52] Dong C Temann UA Flavell RA. Cutting edge: critical role of inducible costimulator in germinal center reactions. J Immunol (2001) 166:3659–62. 10.4049/jimmunol.166.6.3659 11238604

[53] Kobayashi S Hara A Isagawa T Manabe I Takeda K MaruYama T The nuclear IkappaB family protein IkappaBNS influences the susceptibility to experimental autoimmune encephalomyelitis in a murine model. PLoS One (2014) 9:e110838 10.1371/journal.pone.0110838 25347393 PMC4210207

[54] Okamoto K Iwai Y Oh-Hora M Yamamoto M Morio T Aoki K IkappaBzeta regulates T(H)17 development by cooperating with ROR nuclear receptors. Nature (2010) 464:1381–5. 10.1038/nature08922 20383124

[55] Pratama A Srivastava M Williams NJ Papa I Lee SK Dinh XT MicroRNA-146a regulates ICOS-ICOSL signalling to limit accumulation of T follicular helper cells and germinal centres. Nat Commun (2015) 6:6436. 10.1038/ncomms7436 25743066 PMC4366510

[56] Ruan Q Kameswaran V Zhang Y Zheng S Sun J Wang J The Th17 immune response is controlled by the Rel-RORgamma-RORgamma T transcriptional axis. J Exp Med (2011) 208:2321–33. 10.1084/jem.20110462 22006976 PMC3201209

[57] Stone EL Pepper M Katayama CD Kerdiles YM Lai CY Emslie E ICOS coreceptor signaling inactivates the transcription factor FOXO1 to promote Tfh cell differentiation. Immunity (2015) 42:239–51. 10.1016/j.immuni.2015.01.017 25692700 PMC4334393

[58] Warnatz K Bossaller L Salzer U Skrabl-Baumgartner A Schwinger W van der Burg M Human ICOS deficiency abrogates the germinal center reaction and provides a monogenic model for common variable immunodeficiency. Blood (2006) 107:3045–52. 10.1182/blood-2005-07-2955 16384931

[59] Xiao N Eto D Elly C Peng G Crotty S Liu YC. The E3 ubiquitin ligase Itch is required for the differentiation of follicular helper T cells. Nat Immunol (2014) 15:657–66. 10.1038/ni.2912 24859451 PMC4289613

[60] Xiao C Srinivasan L Calado DP Patterson HC Zhang B Wang J Lymphoproliferative disease and autoimmunity in mice with increased miR-17-92 expression in lymphocytes. Nat Immunol (2008) 9:405–14. 10.1038/ni1575 18327259 PMC2533767

[61] Tahiliani V Hutchinson TE Abboud G Croft M Salek-Ardakani S. OX40 cooperates with ICOS To amplify follicular Th cell development and germinal center reactions during infection. J Immunol (2017) 198:218–28. 10.4049/jimmunol.1601356 27895177 PMC5173420

[62] Bartel DP. MicroRNAs: target recognition and regulatory functions. Cell (2009) 136:215–33. 10.1016/j.cell.2009.01.002 19167326 PMC3794896

[63] Ebert MS Sharp PA. Roles for microRNAs in conferring robustness to biological processes. Cell (2012) 149:515–24. 10.1016/j.cell.2012.04.005 22541426 PMC3351105

[64] Mendell JT Olson EN. MicroRNAs in stress signaling and human disease. Cell (2012) 148:1172–87. 10.1016/j.cell.2012.02.005 22424228 PMC3308137

[65] Bronevetsky Y Villarino AV Eisley CJ Barbeau R Barczak AJ Heinz GA T cell activation induces proteasomal degradation of Argonaute and rapid remodeling of the microRNA repertoire. J Exp Med (2013) 210:417–32. 10.1084/jem.20111717 23382546 PMC3570096

[66] Rossi RL Rossetti G Wenandy L Curti S Ripamonti A Bonnal RJ Distinct microRNA signatures in human lymphocyte subsets and enforcement of the naive state in CD4+ T cells by the microRNA miR-125b. Nat Immunol (2011) 12:796–803. 10.1038/ni.2057 21706005

[67] Baumjohann D Ansel KM. MicroRNA-mediated regulation of T helper cell differentiation and plasticity. Nat Rev Immunol (2013) 13:666–78. 10.1038/nri3494 23907446 PMC3980848

[68] Jeker LT Bluestone JA. MicroRNA regulation of T-cell differentiation and function. Immunol Rev (2013) 253:65–81. 10.1111/imr.12061 23550639 PMC3621017

[69] Maul J Baumjohann D. Emerging roles for microRNAs in T follicular helper cell differentiation. Trends Immunol (2016) 37:297–309. 10.1016/j.it.2016.03.003 27068008

[70] Monticelli S. MicroRNAs in T helper cell differentiation and plasticity. Semin Immunol (2013) 25:291–8. 10.1016/j.smim.2013.10.015 24216176

[71] Pagani M Rossetti G Panzeri I de Candia P Bonnal RJ Rossi RL Role of microRNAs and long-non-coding RNAs in CD4(+) T-cell differentiation. Immunol Rev (2013) 253:82–96. 10.1111/imr.12055 23550640

[72] Cobb BS Hertweck A Smith J O’Connor E Graf D Cook T A role for dicer in immune regulation. J Exp Med (2006) 203:2519–27. 10.1084/jem.20061692 17060477 PMC2118134

[73] Muljo SA Ansel KM Kanellopoulou C Livingston DM Rao A Rajewsky K. Aberrant T cell differentiation in the absence of Dicer. J Exp Med (2005) 202:261–9. 10.1084/jem.20050678 16009718 PMC2212998

[74] Baumjohann D Kageyama R Clingan JM Morar MM Patel S de Kouchkovsky D The microRNA cluster miR-17 approximately 92 promotes TFH cell differentiation and represses subset-inappropriate gene expression. Nat Immunol (2013a) 14:840–8. 10.1038/ni.2642 23812098 PMC3720769

[75] Baumjohann D Ansel KM. MicroRNA regulation of the germinal center response. Curr Opin Immunol (2014) 28:6–11. 10.1016/j.coi.2014.01.003 24530656 PMC4037353

[76] Mendell JT. miRiad roles for the miR-17-92 cluster in development and disease. Cell (2008) 133:217–22. 10.1016/j.cell.2008.04.001 18423194 PMC2732113

[77] de Kouchkovsky D Esensten JH Rosenthal WL Morar MM Bluestone JA Jeker LT. MicroRNA-17-92 regulates IL-10 production by regulatory T cells and control of experimental autoimmune encephalomyelitis. J Immunol (2013) 191:1594–605. 10.4049/jimmunol.1203567 23858035 PMC4160833

[78] Kang SG Liu WH Lu P Jin HY Lim HW Shepherd J MicroRNAs of the miR-17 approximately 92 family are critical regulators of TFH differentiation. Nat Immunol (2013) 14:849–57. 10.1038/ni.2648 23812097 PMC3740954

[79] Montoya MM Maul J Singh PB Pua HH Dahlstrom F Wu N A distinct inhibitory function for miR-18a in Th17 cell differentiation. J Immunol (2017) 199:559–69. 10.4049/jimmunol.1700170 28607111 PMC5508756

[80] Wu T Wieland A Lee J Hale JS Han JH Xu X Cutting edge: miR-17-92 is required for both CD4 Th1 and T follicular helper cell responses during viral infection. J Immunol (2015) 195:2515–9. 10.4049/jimmunol.1500317 26276869 PMC5053620

[81] Olive V Li Q He L. mir-17-92: a polycistronic oncomir with pleiotropic functions. Immunol Rev (2013) 253:158–66. 10.1111/imr.12054 23550645 PMC3972423

[82] Baumjohann D. Diverse functions of miR-17-92 cluster microRNAs in T helper cells. Cancer Lett (2018) 423:147–52. 10.1016/j.canlet.2018.02.035 29499238

[83] Jiang S Li C Olive V Lykken E Feng F Sevilla J Molecular dissection of the miR-17-92 cluster’s critical dual roles in promoting Th1 responses and preventing inducible Treg differentiation. Blood (2011) 118:5487–97. 10.1182/blood-2011-05-355644 21972292 PMC3217351

[84] Simpson LJ Patel S Bhakta NR Choy DF Brightbill HD Ren X A microRNA upregulated in asthma airway T cells promotes TH2 cytokine production. Nat Immunol (2014) 15:1162–70. 10.1038/ni.3026 25362490 PMC4233009

[85] Liu SQ Jiang S Li C Zhang B Li QJ. miR-17-92 cluster targets phosphatase and tensin homology and Ikaros family zinc finger 4 to promote TH17-mediated inflammation. J Biol Chem (2014) 289:12446–56. 10.1074/jbc.M114.550723 24644282 PMC4007439

[86] Singh PB Pua HH Happ HC Schneider C von Moltke J Locksley RM MicroRNA regulation of type 2 innate lymphoid cell homeostasis and function in allergic inflammation. J Exp Med (2017) 214:3627–43. 10.1084/jem.20170545 29122948 PMC5716040

[87] Yu D Rao S Tsai LM Lee SK He Y Sutcliffe EL The transcriptional repressor Bcl-6 directs T follicular helper cell lineage commitment. Immunity (2009) 31:457–68. 10.1016/j.immuni.2009.07.002 19631565

[88] Han YC Vidigal JA Mu P Yao E Singh I Gonzalez AJ An allelic series of miR-17 approximately 92-mutant mice uncovers functional specialization and cooperation among members of a microRNA polycistron. Nat Genet (2015) 47:766–75. 10.1038/ng.3321 26029871 PMC4485521

[89] Serr I Furst RW Ott VB Scherm MG Nikolaev A Gokmen F miRNA92a targets KLF2 and the phosphatase PTEN signaling to promote human T follicular helper precursors in T1D islet autoimmunity. Proc Natl Acad Sci U S A (2016) 113:E6659–68. 10.1073/pnas.1606646113 27791035 PMC5087025

[90] Banerjee A Schambach F DeJong CS Hammond SM Reiner SL. Micro-RNA-155 inhibits IFN-gamma signaling in CD4+ T cells. Eur J Immunol (2010) 40:225–31. 10.1002/eji.200939381 19877012 PMC2807623

[91] Haasch D Chen YW Reilly RM Chiou XG Koterski S Smith ML T cell activation induces a noncoding RNA transcript sensitive to inhibition by immunosuppressant drugs and encoded by the proto-oncogene, BIC. Cell Immunol (2002) 217:78–86. 10.1016/S0008-8749(02)00506-3 12426003

[92] Rodriguez A Vigorito E Clare S Warren MV Couttet P Soond DR Requirement of bic/microRNA-155 for normal immune function. Science (2007) 316:608–11. 10.1126/science.1139253 17463290 PMC2610435

[93] Thai TH Calado DP Casola S Ansel KM Xiao C Xue Y Regulation of the germinal center response by microRNA-155. Science (2007) 316:604–8. 10.1126/science.1141229 17463289

[94] Escobar TM Kanellopoulou C Kugler DG Kilaru G Nguyen CK Nagarajan V miR-155 activates cytokine gene expression in Th17 cells by regulating the DNA-binding protein Jarid2 to relieve polycomb-mediated repression. Immunity (2014) 40:865–79. 10.1016/j.immuni.2014.03.014 24856900 PMC4092165

[95] Hu R Huffaker TB Kagele DA Runtsch MC Bake E Chaudhuri AA MicroRNA-155 confers encephalogenic potential to Th17 cells by promoting effector gene expression. J Immunol (2013) 190:5972–80. 10.4049/jimmunol.1300351 23686497 PMC3773482

[96] O’Connell RM Kahn D Gibson WS Round JL Scholz RL Chaudhuri AA MicroRNA-155 promotes autoimmune inflammation by enhancing inflammatory T cell development. Immunity (2010) 33:607–19. 10.1016/j.immuni.2010.09.009 20888269 PMC2966521

[97] Kohlhaas S Garden OA Scudamore C Turner M Okkenhaug K Vigorito E. Cutting edge: the Foxp3 target miR-155 contributes to the development of regulatory T cells. J Immunol (2009) 182:2578–82. 10.4049/jimmunol.0803162 19234151

[98] Lu LF Thai TH Calado DP Chaudhry A Kubo M Tanaka K Foxp3-dependent microRNA155 confers competitive fitness to regulatory T cells by targeting SOCS1 protein. Immunity (2009) 30:80–91. 10.1016/j.immuni.2008.11.010 19144316 PMC2654249

[99] Kuchen S Resch W Yamane A Kuo N Li Z Chakraborty T Regulation of microRNA expression and abundance during lymphopoiesis. Immunity (2010) 32:828–39. 10.1016/j.immuni.2010.05.009 20605486 PMC2909788

[100] Huffaker TB Hu R Runtsch MC Bake E Chen X Zhao J Epistasis between microRNAs 155 and 146a during T cell-mediated antitumor immunity. Cell Rep (2012) 2:1697–709. 10.1016/j.celrep.2012.10.025 23200854 PMC3628775

[101] Rusca N Deho L Montagner S Zielinski CE Sica A Sallusto F miR-146a and NF-kappaB1 regulate mast cell survival and T lymphocyte differentiation. Mol Cell Biol (2012) 32:4432–44. 10.1128/MCB.00824-12 22927641 PMC3486148

[102] Yang L Boldin MP Yu Y Liu CS Ea CK Ramakrishnan P miR-146a controls the resolution of T cell responses in mice. J Exp Med (2012) 209:1655–70. 10.1084/jem.20112218 22891274 PMC3428948

[103] Li B Wang X Choi IY Wang YC Liu S Pham AT miR-146a modulates autoreactive Th17 cell differentiation and regulates organ-specific autoimmunity. J Clin Invest (2017a) 127(10):3702–16. 10.1172/JCI94012 28872459 PMC5617680

[104] Lu LF Boldin MP Chaudhry A Lin LL Taganov KD Hanada T Function of miR-146a in controlling Treg cell-mediated regulation of Th1 responses. Cell (2010) 142:914–29. 10.1016/j.cell.2010.08.012 20850013 PMC3049116

[105] Mohnle P Schutz SV van der Heide V Hubner M Luchting B Sedlbauer J MicroRNA-146a controls Th1-cell differentiation of human CD4+ T lymphocytes by targeting PRKCepsilon. Eur J Immunol (2015) 45:260–72. 10.1002/eji.201444667 25308712

[106] Zhou Q Haupt S Kreuzer JT Hammitzsch A Proft F Neumann C Decreased expression of miR-146a and miR-155 contributes to an abnormal Treg phenotype in patients with rheumatoid arthritis. Ann Rheum Dis (2015) 74:1265–74. 10.1136/annrheumdis-2013-204377 24562503

[107] Zhao JL Rao DS Boldin MP Taganov KD O’Connell RM Baltimore D. NF-kappaB dysregulation in microRNA-146a-deficient mice drives the development of myeloid malignancies. Proc Natl Acad Sci U S A (2011) 108:9184–9. 10.1073/pnas.1105398108 21576471 PMC3107319

[108] Hu R Kagele DA Huffaker TB Runtsch MC Alexander M Liu J miR-155 promotes T follicular helper cell accumulation during chronic, low-grade inflammation. Immunity (2014) 41:605–19. 10.1016/j.immuni.2014.09.015 25367574 PMC4657560

[109] Liu WH Kang SG Huang Z Wu CJ Jin HY Maine CJ A miR-155-Peli1-c-Rel pathway controls the generation and function of T follicular helper cells. J Exp Med (2016) 213(9):1901–19. 10.1084/jem.20160204 27481129 PMC4995083

[110] Crepeau RL Zhang P Usherwood EJ. MicroRNA miR-155 is necessary for efficient gammaherpesvirus reactivation from latency, but not for establishment of latency. J Virol (2016) 90:7811–21. 10.1128/JVI.00521-16 27334594 PMC4988139

[111] Baumjohann D Preite S Reboldi A Ronchi F Ansel KM Lanzavecchia A Persistent antigen and germinal center B cells sustain T follicular helper cell responses and phenotype. Immunity (2013b) 38:596–605. 10.1016/j.immuni.2012.11.020 23499493

[112] Deenick EK Chan A Ma CS Gatto D Schwartzberg PL Brink R Follicular helper T cell differentiation requires continuous antigen presentation that is independent of unique B cell signaling. Immunity (2010) 33:241–53. 10.1016/j.immuni.2010.07.015 20691615 PMC3433066

[113] Linterman MA Vinuesa CG. Signals that influence T follicular helper cell differentiation and function. Semin Immunopathol (2010) 32:183–96. 10.1007/s00281-009-0194-z 20107805

[114] Rolf J Bell SE Kovesdi D Janas ML Soond DR Webb LM Phosphoinositide 3-kinase activity in T cells regulates the magnitude of the germinal center reaction. J Immunol (2010) 185(7):4042–52. 10.4049/jimmunol.1001730 20826752

[115] Jeker LT Zhou X Gershberg K de Kouchkovsky D Morar MM Stadthagen G MicroRNA 10a marks regulatory T cells. PLoS One (2012) 7:e36684. 10.1371/journal.pone.0036684 22629323 PMC3356350

[116] Takahashi H Kanno T Nakayamada S Hirahara K Sciume G Muljo SA TGF-beta and retinoic acid induce the microRNA miR-10a, which targets Bcl-6 and constrains the plasticity of helper T cells. Nat Immunol (2012) 13:587–95. 10.1038/ni.2286 22544395 PMC3499969

[117] Ripamonti A Provasi E Lorenzo M De Simone M Ranzani V Vangelisti S Repression of miR-31 by BCL6 stabilizes the helper function of human follicular helper T cells. Proc Natl Acad Sci U S A (2017) 114:12797–802. 10.1073/pnas.1705364114 29133396 PMC5715737

[118] Li HB Tong J Zhu S Batista PJ Duffy EE Zhao J m(6)A mRNA methylation controls T cell homeostasis by targeting the IL-7/STAT5/SOCS pathways. Nature (2017b) 548:338–42. 10.1038/nature23450 28792938 PMC5729908

[119] Sandberg R Neilson JR Sarma A Sharp PA Burge CB. Proliferating cells express mRNAs with shortened 3’ untranslated regions and fewer microRNA target sites. Science (2008) 320:1643–7. 10.1126/science.1155390 18566288 PMC2587246

[120] Whisenant TC Peralta ER Aarreberg LD Gao NJ Head SR Ordoukhanian P The activation-induced assembly of an RNA/protein interactome centered on the splicing factor U2AF2 regulates gene expression in human CD4 T cells. PLoS One (2015) 10:e0144409. 10.1371/journal.pone.0144409 26641092 PMC4671683

